# Nitric Oxide in the Spinal Cord Is Involved in the Hyperalgesia Induced by Tetrahydrobiopterin in Chronic Restraint Stress Rats

**DOI:** 10.3389/fnins.2021.593654

**Published:** 2021-03-26

**Authors:** Ying Huang, Bo Jiao, Bo Zhu, Bingrui Xiong, Pei Lu, Ling Ai, Ning Yang, Yilin Zhao, Hui Xu

**Affiliations:** ^1^Department of Anesthesiology, Tongji Hospital, Tongji Medical College, Huazhong University of Science and Technology, Wuhan, China; ^2^Department of Anesthesiology, Zhongnan Hospital of Wuhan University, Wuhan, China; ^3^Department of Anesthesiology, Chengdu Second People’s Hospital, Chengdu, China

**Keywords:** tetrahydrobiopterin, stress-induced hyperalgesia, spinal cord, GTP cyclohydrolase 1, inducible nitric oxide synthase

## Abstract

It has been well recognized that exposure to chronic stress could increase pain responding and exacerbate pain symptoms, resulting in stress-induced hyperalgesia. However, the mechanisms underlying stress-induced hyperalgesia are not yet fully elucidated. To this end, we observed that restraint as a stressful event exacerbated mechanical and thermal hyperalgesia, accompanied with up-regulation of nitric oxide (NO) (*P* < 0.001), GTP cyclohydrolase 1 (GCH1) (GCH1 mRNA: *P* = 0.001; GCH1 protein: *P* = 0.001), and tetrahydrobiopterin (BH4) concentration (plasma BH4: *P* < 0.001; spinal BH4: *P* < 0.001) on Day 7 in restraint stress (RS) rats. Intrathecal injection of *N*^ω^-nitro-L-arginine methyl ester (L-NAME), a non-specific NO synthase inhibitor, or *N*-([3-(aminomethyl)phenyl]methyl) ethanimidamide, a special inhibitor of inducible NO synthase (iNOS), for seven consecutive days attenuated stress-induced hyperalgesia and decreased the production of NO (*P* < 0.001). Interestingly, 7-nitro indazole, a special inhibitor of neuronal NO synthase, alleviated stress-induced hyperalgesia but did not affect spinal NO synthesis. Furthermore, intrathecal injection of BH4 not only aggravated stress-induced hyperalgesia but also up-regulated the expression of spinal iNOS (iNOS mRNA: *P* = 0.015; iNOS protein: *P* < 0.001) and NO production (*P* < 0.001). These findings suggest that hyperalgesia induced by RS is associated with the modulation of the GCH1–BH4 system and constitutively expressed spinal iNOS. Thus, the GCH1–BH4–iNOS signaling pathway may be a new novel therapeutic target for pain relief in the spinal cord.

## Introduction

Moderate stress within the range of physiological adaptation could exert beneficial effects on pain alleviation, which has currently been recognized as stress-induced analgesia (SIH). On the contrary, it should be noted that stress also has detrimental actions for pain sensors via eliciting a down-regulation of pain sensitivity, causing the onset of SIH by a wide variety of stress factors, including repeated exposure to the cold environment, restraint, and forced swimming (Satoh et al., 1992; Quintero et al., 2000; da Silva Torres et al., 2003; Imbe et al., 2004, 2010, 2012). Additionally, stress has detrimental effects on several physiological functions, such as affecting behavioral and physiological homeostasis and disturbing neurogenesis, promoting vulnerable susceptibility, and ultimately aggravating damage to neurological systems (Selye, 1976; Kim and Yoon, 1998; McEwen, 1998; Selye, 1998). Several mechanisms have been demonstrated to explain the hyperalgesia induced by chronic stress, including opioid, gamma-aminobutyric acid (GABA), glutamate, monoamine, endocannabinoid, sympathetic adrenomedullary systems, and the hypothalamic–pituitary–adrenal (HPA) axis (Jennings et al., 2014). For instance, the decrease of GABA release and GABA-receptor activation in the spinal cord involves forced swimming SIH and pain-induced c-Fos overexpression (Suarez-Roca et al., 2008; Quintero et al., 2011; Ma et al., 2014). The switch of endogenous opioid signaling from an antinociceptive to a pronociceptive pathway involves chronic SIH (Ferdousi and Finn, 2018). Furthermore, a contribution of the activation of the HPA axis and sympathetic nervous system to SIH has been demonstrated (Elenkov et al., 2000; Golovatscka et al., 2012). Nevertheless, the mechanism underlying SIH has not been fully elucidated.

Tetrahydrobiopterin (BH4) is an essential cofactor for three isoforms of nitric oxide (NO) synthase (NOS): neuronal NOS (nNOS), inducible NOS (iNOS), and endothelial NOS (eNOS). It plays a crucial role in regulating NOS and biosynthesis of NO (Thony et al., 2000; Tegeder et al., 2006; Lam et al., 2007; Werner et al., 2011). Currently, accumulating evidence shows that BH4 is likely to be involved in exacerbating neuropathic and inflammatory pain (Tegeder et al., 2006; Latremoliere et al., 2015), resulting in increased pain sensitivity. Additionally, intrathecal infusion of BH4 could induce and exacerbate nociception by facilitating central sensitization (Nasser et al., 2015). Reducing BH4 production and availability may facilitate the physiological benefits for hyperalgesia in humans. Previous research suggested that BH4 was involved in the pain signaling pathways by regulating neurotransmitters’ biosynthesis, including noradrenaline, adrenaline, dopamine, serotonin, and NO (Thony et al., 2000; Kolinsky and Gross, 2004). Moreover, BH4 induces pain sensitivity partly by the regulation of excess production of NO from nNOS in the L4-5 spinal dorsal root ganglions in the spared nerve injury model of peripheral neuropathic pain (Tegeder et al., 2006), and NO might be involved in the activation of guanylyl cyclase–cGMP–PKG pathway and regulation of the activity of *N*-methyl-D-aspartate (NMDA) receptor (Lipton et al., 1993; Tegeder et al., 2004, 2006).

The biosynthesis of BH4 is highly controlled by three main pathways: the *de novo* synthetic pathway, the salvage pathway, and the cycling pathway. GTP cyclohydrolase 1 (GCH1) is the rate-limiting enzyme responsible for the *de novo* pathway of BH4 synthesis, which could catalyze the initial reaction and convert GTP to 7,8-dihydroneopterin triphosphate (Werner et al., 2011). There is evidence that GCH1 is also involved in developing neuropathic and inflammatory pain. Moreover, intraperitoneal or intrathecal injection (i.t.) of 2,4-diamino-6-hydroxypyrimidine (DAHP), the prototypical GCH1 inhibitor, has antinociceptive effect in the chronic constriction injury (CCI) models and spinal nerve ligation (SNL) models (Tegeder et al., 2006).

Given the critical role of spinal BH4 in hyperalgesia, we aimed to investigate whether SIH is associated with the GCH1–BH4–NO system. To this end, we first established the chronic restraint stress (RS) model to investigate its effects on pain intensity and clarify its relationship with spinal GCH1. We further designed to observe whether BH4 in SIH is mediated mainly by NO. Finally, the present study using a specific NOS inhibitor to validate NO comes from which NOS isoform mainly modulates pain responses. Collectively, our data for the first time implicate the role of spinal BH4 in hyperalgesia.

## Results

### GTP Cyclohydrolase 1–Tetrahydrobiopterin Axis Was Involved in the Development and Maintenance of Stress-Induced Analgesia

#### Chronic Restraint Stress-Induced Time-Dependent Mechanical and Thermal Hyperalgesia

**FIGURE 1:**
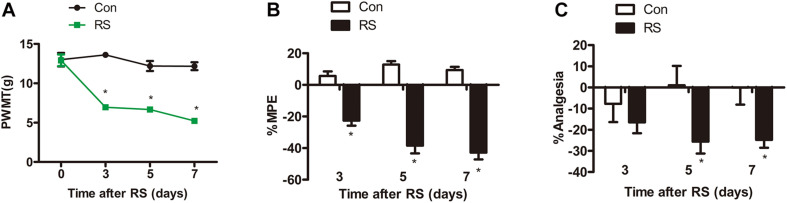
Mechanical or thermal nociceptive thresholds in rats with or without chronic RS. **(A–C)** Nociceptive behavior tests including PWMT (RS effect: *F*_1,22_ = 192.322, *P* < 0.001; observation intervals: *F*_3,66_ = 22.641, *P* < 0.001; interaction: *F*_3,66_ = 16.798, *P* < 0.001), PWTL (RS effect: *F*_1,22_ = 141.084, *P* < 0.001; observation intervals: *F*_2,44_ = 4.606, *P* = 0.015; interaction: *F*_2,44_ = 12.632, *P* < 0.001), and TFL (RS effect: *F*_1,22_ = 5.457, *P* = 0.029; observation intervals: *F*_2,44_ = 0.002, *P* = 0.998; interaction: *F*_2,44_ = 2.552, *P* = 0.089). Data are shown as mean ± SEM (*n* = 12). *Significant difference with respect to control groups (two-way ANOVA with repeated measures in nociceptive behavior tests, followed by Bonferroni *post hoc* test or Dunnett’s T3 test if necessary). *P < 0.05. RS, restraint stress; PWMT, paw withdrawal mechanical threshold; PWTL, paw withdrawal thermal latency; TFL, tail-flick latency; NO, nitric oxide; iNOS, inducible nitric oxide synthase.

Paw withdrawal mechanical threshold (PWMT) test, paw withdrawal thermal latency (PWTL), and tail-flick latency (TFL) were commonly used to evaluate pain sensitivity (Sung et al., 2004; Guan et al., 2015; Deciga-Campos et al., 2016). All tests were conducted on the first day before establishing the RS model and on the third, fifth, and seventh days. Before the model was established, both left hind paws’ mechanical pain sensitivity and thermal pain sensitivity had no statistical difference (*P* > 0.05). On the third, fifth, and seventh days of RS, the PWMT of the RS rats was significantly decreased to 6.94 ± 1.24, 6.67 ± 0.9, and 5.22 ± 0.94 g, respectively (RS effect: *F*_1,22_ = 192.322, *P* < 0.001; observation intervals: *F*_3,66_ = 22.641, *P* < 0.001; interaction: *F*_3,66_ = 16.798, *P* < 0.0001, Figure 1A), suggesting that RS has been successfully developed as a model of mechanical tactile allodynia. As to the PWTL, the increase in %MPE was significantly decreased in RS rats compared with that of age-matched control rats (Figure 1B), which represented time-dependent thermal hyperalgesia. Compared with the control rats, %Analgesia in the RS group was significantly decreased on Day 3, Day 5, and Day 7, while %Analgesia of rats subjected to RS was slightly decreased on Day 5, and Day 7 compared with Day 3 (RS effect: *F*_1,22_ = 5.457, *P* = 0.029; observation intervals: *F*_2,44_ = 0.002, *P* = 0.998; interaction: *F*_2,44_ = 2.552, *P* = 0.089, Figure 1C). Therefore, it is likely that the RS models and their related SIH, including abnormality in mechanical allodynia and thermal hyperalgesia, were successfully established.

#### Chronic Restraint Stress-Induced Hypomethylation of GTP Cyclohydrolase 1, Thus Up-Regulating the Expression of Spinal GTP Cyclohydrolase 1

**FIGURE 2:**
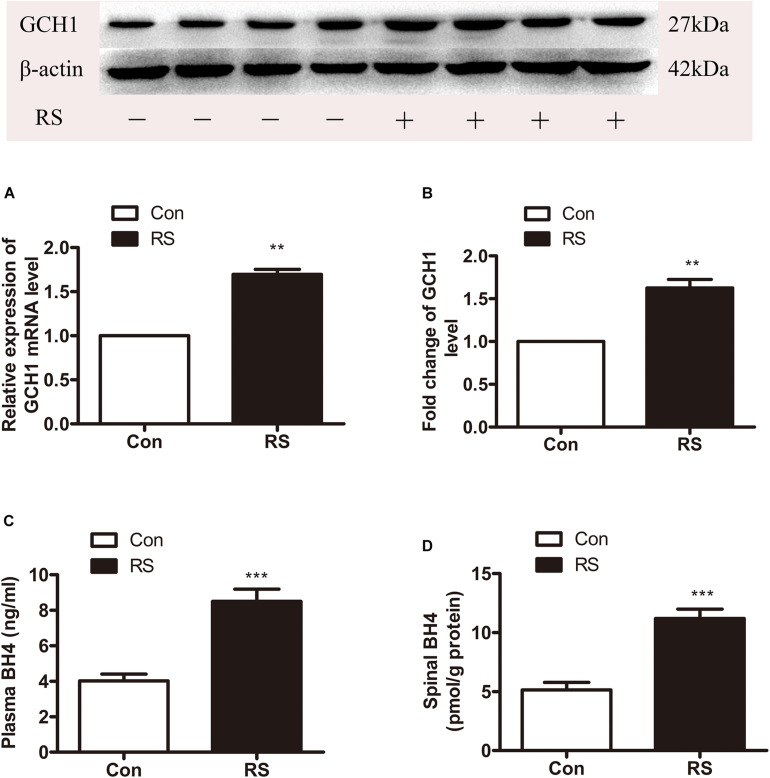
The expression of spinal GCH1 and the production of plasma and spinal BH4 in the rats with or without chronic RS. **(A,B)** The expression of spinal GCH1 mRNA (*P* = 0.001) and spinal GCH1 protein (*P* = 0.001) in the RS rats and control rats. **(C)** The concentration of plasma BH4 (*P* < 0.001) and spinal BH4 (*P* < 0.001) in the RS and control rats. *Significant difference with respect to control groups (Student’s t test). **P < 0.01 and ***P < 0.001. GCH1, GTP cyclohydrolase 1; BH4, tetrahydrobiopterin; RS, restraint stress.

DNA methylation is an essential mechanism for the control gene, mainly in the CpG islands (CGIs). We analyzed the CGI across the whole GCH1 gene (i.e., 5,000 bp upstream of the first exon to 1,000 bp downstream of the last exon) using the CGI prediction software because of no available annotation for the transcription of the start and end of the rats GCH1. Programs #21 and #22 (Table 1) were the recommendations, and a total of 77 CGIs were investigated. As we expected, RS rats represented a hypomethylation of GCH1 in the location of CpG 14, 15 (27909945, 27909939) and CpG 39 (27909657) compared with the control rats (CpG 14, 15: *P* < 0.001; CpG 39: *P* = 0.016, Table 2). Moreover, GCH1 demethylation induced the activation and expression of GCH1 mRNA and protein (GCH1 mRNA: *P* = 0.001; GCH1 protein: *P* = 0.001, Figures 2A,B).

**TABLE 1:** Primer design.

Program #21	Primer	Start	Size	Tm	GC%	C’s
	5′ primer	4811	24	59.60	33	5
	3′ primer	5166	25	60.47	40	4
Program #22	5′ primer	5142	25	60.47	40	4
	3′ primer	5719	24	60.17	29	8
Program #21 Product size: 356 No. of CPGs: 32 Coverage: 31						
Program #22 Product size: 578 No. of CPGs: 45 Coverage: 41						

*Program #21: 5′ primer sequence: aggaagagagGGTTTAATTTGAGGGTTGTTTTGT; 3′ primer sequence: cagtaatacgactcactatagggagaaggctAAATAAAAAAATCCCACATTCCCT. Program #22: 5′ primer sequence: aggaagagagGGGTGTAAGGTGTATTAATGGGTTT; 3′ primer sequence: cagtaatacgactcactatagggagaaggct AAATAAAAAAATCCCACATTCCCT.*

**TABLE 2:** GCH1 DNA methylation values for each CpG unit in control and RS groups.

CpG sites	Position	Con (*n* = 6)	RS (*n* = 6)	*P*-value
CpG 14, 15	27909945; 27909939	0.2233 ± 0.04082	0.04457 ± 0.01820	**0.001
CpG 39	27909657	0.4783 ± 0.14932	0.2350 ± 0.14349	*0.016

All data are presented as the mean ± SEM.*P*-values were calculated with Student’s test, and *P* < 0.05 was considered statistically significant.

*P < 0.05.

**P < 0.001.RS, restraint stress.

#### Effects of Chronic Restraint Stress on the Biosynthesis of Spinal and Plasma Tetrahydrobiopterin

GTP cyclohydrolase 1 is the first rate-limiting enzyme of BH4 in the *de novo* synthesis; thus, the GCH1 gene’s demethylation and activation of GCH1 mRNA and protein might improve the biosynthesis of BH4. Therefore, the plasma and spinal BH4 were detected by high-performance liquid chromatography (HPLC). As demonstrated in Figures 2C,D, the content of BH4 in the plasma was significantly increased to the spinal BH4 compared with the control rats (plasma BH4: *P* < 0.001; spinal BH4: *P* < 0.001).

#### Chronic Restraint Stress-Induced Analgesia Was Attenuated by DAHP and Exacerbated by Intrathecal Injection of Tetrahydrobiopterin Into the Spinal Cord

**FIGURE 3:**
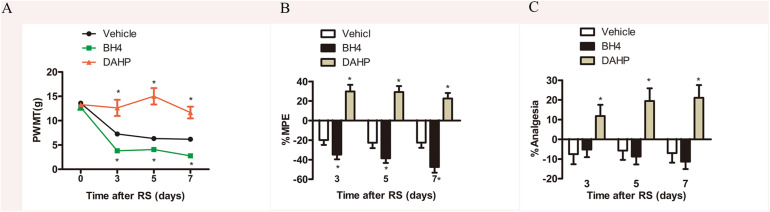
Effects of either DAHP or BH4 intrathecal administration on nociceptive behavior in rats subjected to chronic RS. Chronic RS rats were consecutively administered with either DAHP (6 mg/kg) or BH4 (1 μg/ml, 10 μl) as the study protocol in Figure 1. **(A–C)** Nociceptive behavior tests including PWMT (treatment: *F*_2,23_ = 73.995, *P* < 0.001; observation intervals: *F*_3,69_ = 45.069, *P* < 0.001; interaction: *F*_6,69_ = 11.544, *P* < 0.0001); **(B)** treatment: *F*_2,23_ = 51.543, *P* < 0.001; observation intervals: *F*_2,46_ = 3.942, *P* = 0.026; interaction: *F*_4,46_ = 0.761, *P* = 0.556; **(C)** treatment: *F*_2,23_ = 50.134, *P* < 0.001; observation intervals: *F*_2,46_ = 6.665, *P* = 0.003; interaction: *F*_4,46_ = 0.257, *P* = 0.904. Data are shown as mean ± SEM (*n* = 8–10). *Significant difference with respect to vehicle groups (two-way ANOVA with repeated measures in nociceptive behavior tests, followed by Bonferroni *post hoc* test or Dunnett’s T3 test if necessary, *P < 0.05). BH4, tetrahydrobiopterin; RS, restraint stress; PWMT, paw withdrawal mechanical threshold.

To further investigate the functional role of spinal GCH1 and BH4 in SIH, rats were intrathecally injected with either BH4 or DAHP 15 min prior to RS, respectively. As shown in Figures 3A–C, on Days 3, 5, and 7, the specific inhibitor of GCH1 DAHP (6 mg/kg, i.t.) presented an analgesic effect in RS rats by evaluation of the PWMT, PWTL, and TFT in the same hind paws compared with the vehicle-treated rats (PWMT: treatment: *F*_2,23_ = 73.995, *P* < 0.001; observation intervals: *F*_3,69_ = 45.069, *P* < 0.001; interaction: *F*_6,69_ = 11.544, *P* < 0.0001; %MPE: treatment: *F*_2,23_ = 51.543, *P* < 0.001; observation intervals: *F*_2,46_ = 3.942, *P* = 0.026; interaction: *F*_4,46_ = 0.761, *P* = 0.556; %Analgesia: treatment: *F*_2,23_ = 50.134, *P* < 0.001; observation intervals: *F*_2,46_ = 6.665, *P* = 0.003; interaction: *F*_4,46_ = 0.257, *P* = 0.904). Interestingly, BH4 caused a rapid and long-lasting increase of PWMT and PWTL in RS rats. DAHP (6 mg/kg, i.t.) failed to elicit any pharmacological effects on the mechanical or heat pain sensitivity in control rats, while BH4 (1 μg/μl, 10 μl, i.t.) evoked a hyperalgesia in control rats (data not shown). One plausible explanation for this discrepancy is that in healthy animals, the activity of sensory neurons in *de novo* pathway is lower, while the recycling and salvage pathways maintain the basal homeostatic BH4. However, after nerve injury or inflammation, the activation and expression of GCH1 are remarkably up-regulated in sensory neurons, thus inducing an overproduction of BH4 (Tegeder et al., 2006), suggesting that BH4 may possess pronociceptive properties at central sites of the somatosensory system.

Based on our findings, we concluded that the GCH1–BH4 system was involved in the occurrence and development of hyperalgesia induced by chronic RS. Moreover, inhibition of the *de novo* synthesis of BH4 by blocking GCH1 might be a new therapeutic target for chronic pain.

### The Role of Spinal Nitric Oxide in the Development of Stress-Induced Analgesia Induced by Tetrahydrobiopterin

**FIGURE 4:**
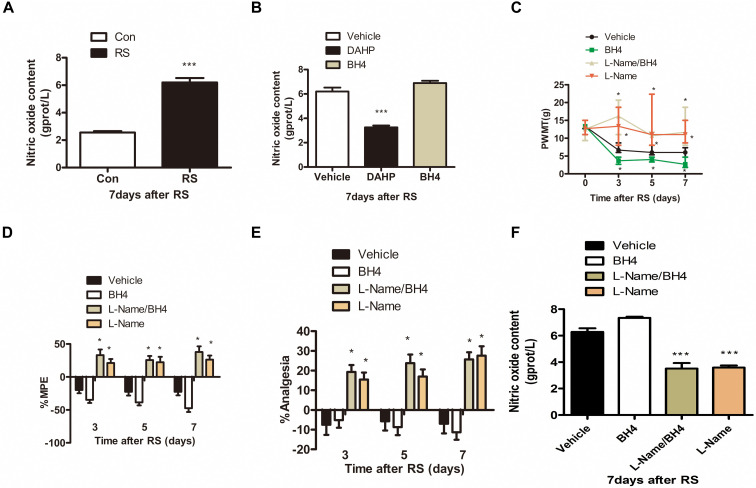
The expression of spinal NO in the rats subjected or not subjected to chronic RS and effect of L-NAME on the nociceptive behavior and spinal NO in the rat models of RS. **(A)** The expression of spinal NO in the rats subjected or not subjected to chronic RS, *P* < 0.001. **(B)** Effects of either DAHP or BH4 intrathecal administration on spinal NO in rats subjected to chronic RS. Chronic RS rats received chronic intrathecal treatment with either DAHP (6 mg/kg) or BH4 (1 μg/ml, 10 μl) from the first day of establishing chronic RS until the end of the experiment, *F*_2,15_ = 67.94, *P* < 0.001. **(C–E)** Effect of L-NAME intrathecal administration on nociceptive behavior in rats subjected to chronic RS; chronic RS rats that received chronic intrathecal treatment with L-NAME (30 μg/μl, 10 μl) 30 min prior to BH4 (1 μg/ml, 10 μl), L-NAME (30 μg/μl, 10 μl), and BH4 (1 μg/ml, 10 μl) treatment. Nociceptive behavior tests including PWMT (treatment: *F*_3,33_ = 47.94, *P* < 0.001; observation intervals: *F*_3,99_ = 49.132, *P* < 0.001; interaction: *F*_9,99_ = 16.453, *P* < 0.0001), PWTL (treatment: *F*_3,33_ = 34.594, *P* < 0.001, observation intervals: *F*_2,66_ = 1.131, *P* = 0.329; interaction: *F*_6,66_ = 3.15, *P* = 0.009), and TFL (treatment: *F*_3,33_ = 22.943, *P* < 0.001; observation intervals: *F*_2,66_ = 1.598, *P* = 0.21; interaction: *F*_6,66_ = 2.904, *P* = 0.014). **(F)** Effect of intrathecal administration of L-NAME on the expression of spinal NO, *F*_3,20_ = 51.906, *P* < 0.001. *Significant difference with respect to control or vehicle groups (two-way ANOVA with repeated measures). *P < 0.05 and ***P < 0.001. NO, nitric oxide; RS, restraint stress; L-NAME, *N*^ω^-nitro-L-arginine methyl ester; NO, nitric oxide; BH4, tetrahydrobiopterin; PWMT, paw withdrawal mechanical threshold; PWTL, paw withdrawal thermal latency; TFL, tail-flick latency.

Considerable evidence has shown that NO has an important role in the peripheral and central nervous system and participates in a wide variety of physiologic and pathophysiologic processes, such as neurotoxicity and pathologic pain (Meller et al., 1992; Wong et al., 1998; Tang et al., 2007). Thus, we detected spinal NO expression in the RS and control rats to validate our hypothesis that NO might be involved in the SIH. As expected, RS up-regulated spinal NO expression compared with the control rats (*P* < 0.001, Figure 4A). To determine whether DAHP decreased spinal NO expression, we also detected spinal NO in the DAHP-treated and BH4-treated rats. As shown in Figure 4B, the expression of spinal NO in the DAHP group was robustly decreased compared with that of the vehicle RS rats, while it is slightly increased in the BH4 group (*F*_2,15_ = 67.94, *P* < 0.001). Based on our findings, we supposed that the effect of BH4 in SIH was regulated by spinal NO. Therefore, a non-specific inhibitor of NO, *N*^ω^-nitro-L-arginine methyl ester (L-NAME), was intrathecally injected into RS rats to test our hypothesis. Both L-NAME (30 μg/μl, 10 μl, i.t.) and BH4 (1 μg/μl, 10 μl, i.t.) were intrathecally injected into the RS rats, and L-NAME was administrated 30 min before BH4. Low pain sensitivity was induced in rats treated with either L-NAME/BH4 or L-NAME as compared with the vehicle-treated rats and BH4-treated rat (PWMT: treatment: *F*_3,33_ = 47.94, *P* < 0.001; observation intervals: *F*_3,99_ = 49.132, *P* < 0.001; interaction: *F*_9,99_ = 16.453, *P* < 0.001; %MPE: treatment: *F*_3,33_ = 34.594, *P* < 0.001; observation intervals: *F*_2,66_ = 1.131, *P* = 0.329; interaction: *F*_6,66_ = 3.15, *P* = 0.009; %Analgesia: treatment: *F*_3,33_ = 22.943, *P* < 0.001; observation intervals: *F*_2,66_ = 1.598, *P* = 0.21; interaction: *F*_6,66_ = 2.904, *P* = 0.014, Figures 4C–E). Furthermore, spinal NO expression was significantly decreased in the L-NAME/BH4 group and L-NAME group compared with the vehicle group (*F*_3,20_ = 51.906, *P* < 0.001, Figure 4F). Interestingly, BH4 intrathecally injected rats only presented a slight increase of spinal NO. This might be explained by the fact that RS induced a marked biosynthesis of BH4, and endogenous BH4 catalyzed NOS to produce NO, reaching the peak.

### Inducible Nitric Oxide Synthase–Nitric Oxide Cascade System Modulated the Role of Tetrahydrobiopterin in the Stress-Induced Analgesia in the Spinal Cord

**FIGURE 5:**
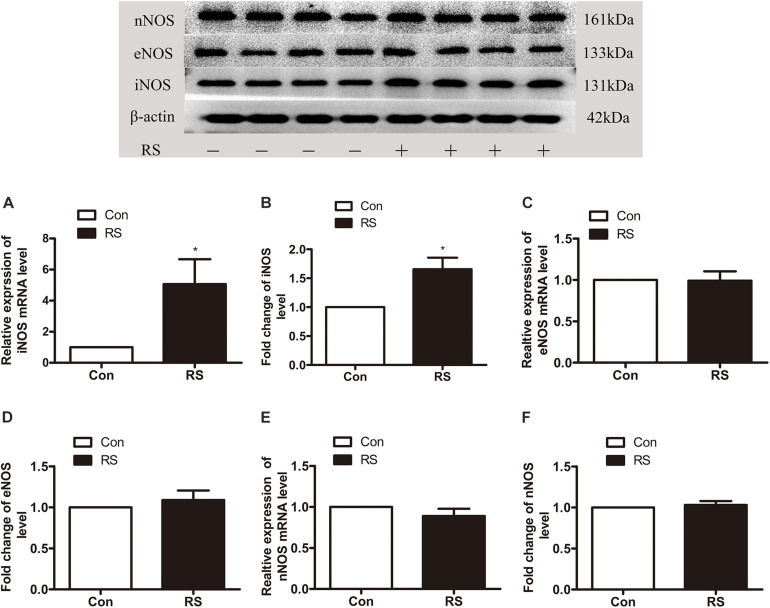
The expression of three isoforms of NOS in the spinal cord in the chronic RS or control rats. **(A,B)** The expression of spinal iNOS mRNA (*P* = 0.045) and protein (*P* = 0.046) in the rats subjected or not subjected to chronic RS. **(C,D)** The expression of spinal eNOS mRNA (*P* = 0.937) and protein (*P* = 0.449) in the rats subjected or not subjected to chronic RS. **(E,F)** The expression of spinal nNOS mRNA (*P* = 0.3) and protein (*P* = 0.565) in the RS rats or control rats. Data are presented as mean ± SEM (*n* = 4), *P < 0.05. NOS, nitric oxide synthase; RS, restraint stress; iNOS, inducible nitric oxide synthase; eNOS, endothelial nitric oxide synthase; nNOS, neuronal nitric oxide synthase.

**FIGURE 6:**
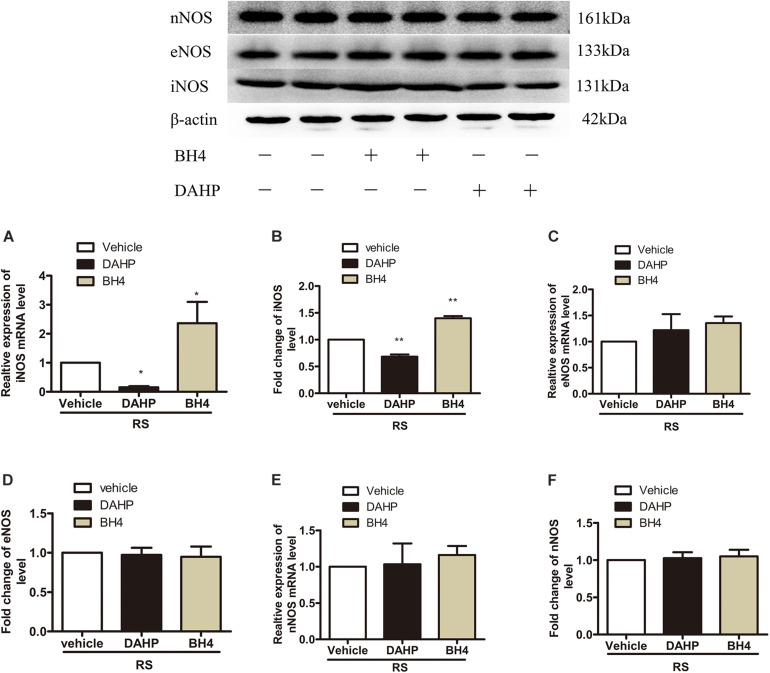
Effect of either DAHP or BH4 intrathecal administration on the expression of three spinal NOS isoforms. **(A,B)** The expression of spinal iNOS mRNA (*F*_2,9_ = 6.869, *P* = 0.015) and protein (*F*_2,9_ = 123.036, *P* < 0.001) in rats treated with DAHP or BH4. **(C,D)** The expression of spinal eNOS mRNA (*F*_2,9_ = 0.879, *P* = 0.448) and protein (*F*_2,9_ = 0.081, *P* = 0.923) in rats treated with DAHP or BH4. **(E,F)** The expression of spinal nNOS mRNA (*F*_2,9_ = 0.223, *P* = 0.804) and protein (*F*_2,9_ = 0.131, *P* = 0.879) in rats treated with DAHP or BH4. Data are shown as mean ± SEM (*n* = 4). *Significant difference with respect to vehicle groups (one-way ANOVA followed by Bonferroni *post hoc* test or Dunnett’s T3 test if necessary). *P < 0.05 and **P < 0.01. BH4, tetrahydrobiopterin; NOS, nitric oxide synthase; iNOS, inducible nitric oxide synthase; eNOS, endothelial nitric oxide synthase; nNOS, neuronal nitric oxide synthase.

**FIGURE 7:**
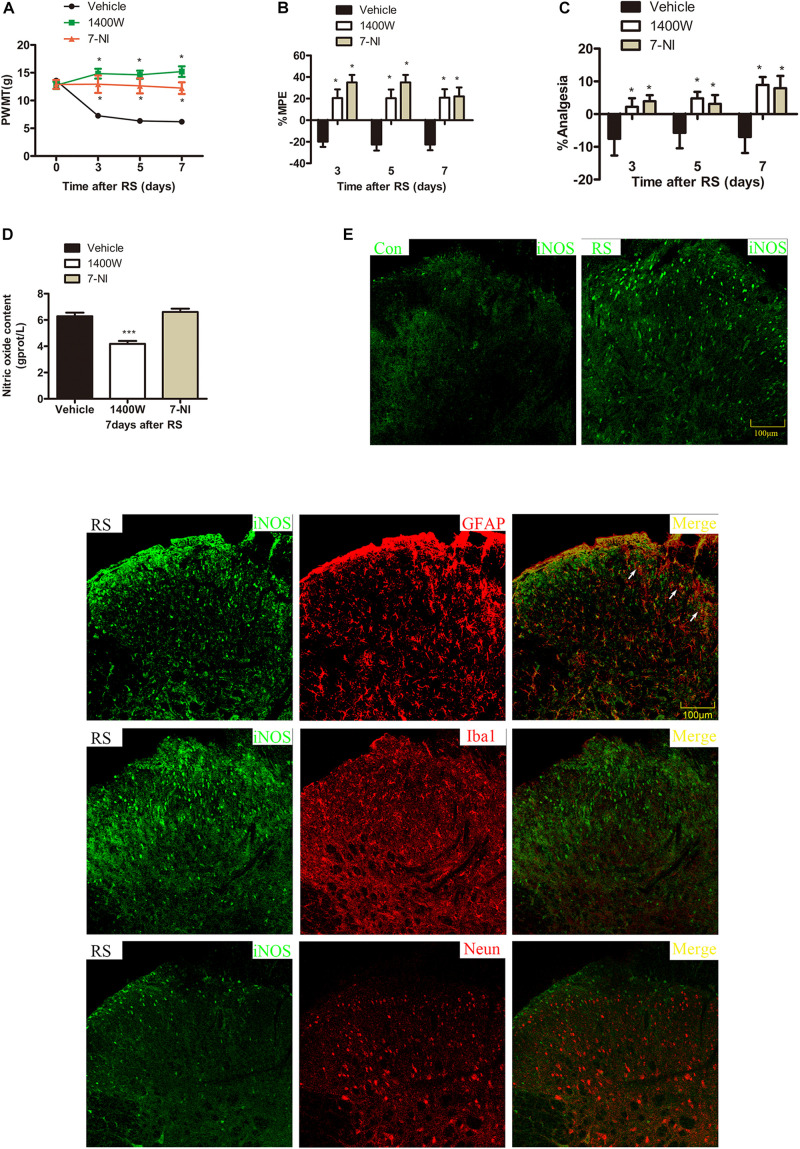
Effect of specific 1400 W or 7-NI on nociceptive behavior and spinal NO in rats subjected to chronic RS, and the cell type specificity of iNOS. Chronic RS rats were consecutively treated with either 1400 W (1 μg/ml, 10 μl) or 7-NI (40 μg/ml, 10 μl). **(A–C)** Nociceptive behavior tests including PWMT (treatment: *F*_2,25_ = 20.961, *P* < 0.001; observation intervals: *F*_3,75_ = 4.045, *P* = 0.016; interaction: *F*_6,75_ = 9.247, *P* < 0.0001), PWTL (treatment: *F*_2,25_ = 15.561, *P* < 0.001; observation intervals: *F*_2,50_ = 2.038, *P* = 0.154; interaction: *F*_4,50_ = 1.831, *P* = 0.158), and TFL (treatment: *F*_2,25_ = 4.898, *P* = 0.016; observation intervals: *F*_2,50_ = 3.174, *P* = 0.064; interaction: *F*_4,50_ = 1.1, *P* = 0.362). **(D)** Effect of 1400 W or 7-NI on the expression of spinal NO, *F*_2,15_ = 27.36, *P* < 0.001. Data are shown as mean ± SEM (*n* = 8–10). *Significant difference with respect to vehicle groups (two-way ANOVA with repeated measures in nociceptive behavior tests and one-way ANOVA in spinal NO measurement followed by Bonferroni *post hoc* test or Dunnett’s T3 test if necessary), *P < 0.05 and ***P < 0.001. **(E)** In the superficial layer of the spinal dorsal horn (lamina I–III), iNOS-positive cells were increased in the RS rats compared with control rats. **(F)** Confocal images of iNOS immunostaining (green) and its colocalization with astrocytes (GFAP, red), but not with microglia (Iba1, red) or neurons (Neun, red) in the superficial spinal dorsal horns (lamina I–III, *n* = 3 in each group). Scale bar = 100 μm. 7-NI, 7-nitro indazole; NO, nitric oxide; RS, restraint stress; iNOS, inducible nitric oxide synthase; PWMT, paw withdrawal mechanical threshold; PWTL, paw withdrawal thermal latency; iNOS, inducible nitric oxide synthase.

To further investigate which isoform NOS mainly modulates the role of BH4 in the SIH in the spinal cord, nNOS, iNOS, and eNOS were subsequently examined. The expression level of spinal iNOS in the RS group was remarkably up-regulated as compared with the control group rats (iNOS mRNA: *P* = 0.045, iNOS protein: *P* = 0.046, Figures 5A,B), while no significant differences of spinal nNOS and eNOS were observed between the RS group and control group (nNOS mRNA: *P* = 0.3; nNOS protein: *P* = 0.565; eNOS mRNA: *P* = 0.937; eNOS protein: *P* = 0.449, Figures 5C–F). Furthermore, the expression of spinal iNOS was remarkably up-regulated in the rat models of RS treated with BH4; however, it was distinctively down-regulated by DAHP (iNOS mRNA: *F*_2,9_ = 6.869, *P* = 0.015; iNOS protein: *F*_2,9_ = 123.036, *P* < 0.001, Figures 6A,B), which was consistent with previous behavioral results. On the other hand, compared with those in the vehicle group, spinal nNOS and eNOS were neither down-regulated in the DAHP-treated group nor up-regulated in the BH4-treated group in the rat model of RS (nNOS mRNA: *F*_2,9_ = 0.223, *P* = 0.804; nNOS protein: *F*_2,9_ = 0.131, *P* = 0.879; eNOS mRNA: *F*_2,9_ = 0.879, *P* = 0.448; eNOS protein: *F*_2,9_ = 0.081, *P* = 0.923, Figures 6C–F). Based on our findings, we might conclude that NO comes from the iNOS isoform mainly modulating the role of BH4 in the SIH in the spinal cord. To further validate our hypothesis, the specific inhibitor of nNOS [7-nitro indazole (7-NI), 40 μg/μl, 10 μl, i.t.] and iNOS (1400W, 1 μg/μl, 10 μl, i.t.) was subsequently administrated. As we expected, 1400 W effectively alleviated the SIH by inhibiting the iNOS–NO cascade system in the spinal cord. Interestingly, 7-NI attenuated the SIH, while it had no statistical effect on the spinal NO (PWMT: treatment: *F*_2,25_ = 20.961, *P* < 0.001; observation intervals: *F*_3,75_ = 4.045, *P* = 0.016; interaction: *F*_6,75_ = 9.247, *P* < 0.001; %MPE: treatment: *F*_2,25_ = 15.561, *P* < 0.001; observation intervals: *F*_2,50_ = 2.038, *P* = 0.154; interaction: *F*_4,50_ = 1.831, *P* = 0.158; %Analgesia: treatment: *F*_2,25_ = 4.898, *P* = 0.016; observation intervals: *F*_2,50_ = 3.174, *P* = 0.064; interaction: *F*_4,50_ = 1.1, *P* = 0.362; NO: *F*_2,15_ = 27.36, *P* < 0.001, Figures 7A–D). These findings indicate that 1400 W through inhibiting the iNOS–NO cascade system to mainly modulate the role of BH4 in the SIH in the spinal cord. Furthermore, results of iNOS-immunoreactive intensity were consistent with the results of western blot and qRT-PCR analysis (Figure 7E). To the best of our knowledge, there are no other studies reporting on the cellular localization of iNOS in the spinal cord in RS rats. Therefore, double immunofluorescence of iNOS was performed with different cell markers, including GFAP (astrocyte biomarker), Iba1 (microglia biomarker), and NeuN (neuron biomarker). Spinal samples acquired from the RS rats indicated that iNOS was co-expressed with astrocytes in the superficial layer of the dorsal horn of the spinal cord, instead of microglia and neurons. These results indicate that iNOS induced by chronic RS in the spinal cord is produced by astrocytes (Figure 7F).

## Discussion

Chronic stress could enhance pain sensitivity and induce hyperalgesia through the supraspinal pain conduction pathway [including the cerebral cortex, amygdala, periaqueductal gray, and rostral ventromedial medulla (RVM)] and spinal dorsal horn (Jennings et al., 2014; Wippert and Wiebking, 2018). Nociceptive stimulation is transmitted to the somatosensory cortex through the ascending pain pathway. Subsequently, descending facilitatory or inhibitory pathways are considered to be activated to enhance or restrain nociceptive transmission, respectively. The descending projections of RVM to the spinal dorsal horn play a key role in SIH (Jennings et al., 2014). Although the mechanism in the spinal cord involved in the SIH remains elusive, previous studies have demonstrated that the occurrence and maintenance of different pain share a common signal transduction pathway of BH4 in various pain models (Tegeder et al., 2006; Kim et al., 2009; Latremoliere and Costigan, 2011). Nevertheless, there is no evidence showing that no injury stress-induced hyperalgesia is dependent on BH4. In the present study, our results demonstrated that (i) chronic RS could induce mechanical allodynia and thermal hyperalgesia in the rat model; (ii) the gene of GCH1 demethylation was concomitant with the up-regulation of spinal GCH1 mRNA and protein in the chronic RS rats; (iii) chronic RS-induced hyperalgesia might be partly regulated by the overproduction of NO, accompanied with BH4; and (iv) the activation of iNOS–NO cascade system in the spinal cord partly modulated the SIH. In general, our study first implicates a critical role of BH4 in the occurrence and development of SIH, but the precise mechanisms are not clear.

GTP cyclohydrolase 1, the first rate-limiting enzyme in the *de novo* synthesis of BH4, has been implicated in developing and maintaining neuropathic and inflammatory pain (Nasser and Moller, 2014). Specifically, we found an up-regulation of GCH1 mRNA, accompanied by increased protein expression in the chronic RS on 7 days. In support of this, demethylation of gene GCH1 elicited by repeated exposure to RS was observed. This study is the first to validate the correlation between SIH development and GCH1 methylation in preclinical studies. Accumulating evidence has demonstrated that DNA methylation is an essential modification of protein and nucleic acid. It could modulate the expression and closure of genes closely associated with many diseases (e.g., cancer). Thus, it is a critical component of epigenetic machinery (Wang et al., 2016). Tegeder et al. (2006) have declared that single-nucleotide polymorphisms (SNPs) in the gene for the GCH1 alter responses to noxious stimuli in healthy humans and susceptibility to neuropathic pain in patients. Nevertheless, the precise locations mediating the regulation of GCH1 transcription are not determined in their study. For the first time, our study elucidates that three CGIs (27909945, 27909939, and 27909657) regulate it, providing a function gene therapy for chronic pain. To determine if blocking GCH1 attenuates pain sensitivity, we intrathecally administrated GCH1 unique inhibitor DAHP (6 mg/kg) via a lumbar spinal catheter. As expected, DAHP effectively attenuated mechanical allodynia and thermal hyperalgesia in rats exposed to RS. Interestingly, unlike stress-elicited pain conditions, DAHP did not influence behavioral responses to stimuli, consistent with the previous study (Tegeder et al., 2006). A previous observation has demonstrated that DAHP-mediated inhibition of GCH1 may be GFRP-dependent, and DAHP could selectively inhibit GCH1 activity via competition for substrate GTP (Kolinsky and Gross, 2004). However, in the prevention study, the precise mechanisms of DAHP inhibition GCH1 required clarity in further studies. Furthermore, it has been established that GCH1 activity is subjected to feedback inhibition by the *de novo* synthesis end product BH4. Besides, BH4 has been implicated to play a crucial role in the distinct peripheral inflammatory pain and neuropathic pain models. Based on these previous findings, we designed to detect the level of plasma and spinal BH4 in rats exposed to chronic RS to validate whether no injury stress-induced hyperalgesia is dependent on the BH4 pathway. Following the procedure of RS, both the plasma BH4 and spinal BH4 concentrations were increased. To further determine whether BH4 could exacerbate pain sensitivity, we intrathecally injected its active enantiomer 6(*R*)-5,6,7,8-BH4 dihydrochloride. Consistent with the previous reports and our assumption, intrathecal administration of BH4 at a dose of 10 μg enhanced mechanical allodynia and thermal hyperalgesia in stressed rats and produced a rapid and long-lasting pain in normal control rats. To sum up, these findings reasonably suggest that the BH4 pathway is associated with the SIH and GCH1 and might be a function gene therapy for chronic pain. Nevertheless, the underlying mechanisms of BH4 modulating the development and maintenance of hyperalgesia are intriguing issues to clarify in further studies.

Studies from the last decades also demonstrated that in either the central mechanisms or the peripheral mechanisms do distinct neurotransmitters (e.g., norepinephrine, 5-hydroxytryptamine, 5-hydroxyindoleacetic acid, dopamine, and NO) play a crucial fundamental role during the nociceptive afferent and descending pain facilitation (Khasar et al., 2009; Kumar et al., 2010; Donello et al., 2011). Several evidence lines have demonstrated that BH4 is an essential cofactor for tyrosine hydroxylase, phenylalanine hydroxylase, tryptophan hydroxylase, and three distinct isoforms of NOS (Nandi et al., 2005; Vyas-Read et al., 2007). Based on the previous studies, we reasonably speculated that BH4 is the common messenger to mediate these neurotransmitters in the SIH pathway. Notably, we observed robust increases of nitrite and nitrate, widely represented as NO production indicators in stressed rats. Furthermore, to validate whether BH4 enhances nociceptive responses partly through the NO pathway, we detected the expression of spinal NO metabolites in the BH4- and DAHP-treated stressed rats. Consistent with our hypothesis, NO metabolites were significantly decreased after DAHP treatment. Interestingly, only a slight increase of NO metabolites was observed after the administration of BH4 as compared with those in the vehicle rats, which is not consistent with the previous report (Tegeder et al., 2006). The controversial results might be due to the different preclinical models and the time point of observation. L-NAME, a non-selective inhibitor of NO, was widely injected in various pain models, and it effectively attenuated the pain threshold. Our studies demonstrated that pretreatment with L-NAME did attenuate the effect of BH4 on nociceptive responses in stressed rats. Therefore, it is reasonable to clarify that nociceptive effects of BH4 may be exerted via facilitating central sensitization under chronic pain conditions and implicate a vital role of NO pathway in the duration of hyperalgesia in chronic RS (Nasser et al., 2015). Particularly, hyperalgesia effect of NO by facilitating nociceptive transmission might be mediated by the release of glutamate, activation of NMDA glutamate receptor (which increased the c-fos expression and NO synthesis), activation of the guanylyl cyclase-cyclic GMP-PKG pathway, and the phosphorylation of MAP kinase (such as p38 and ERK) at the spinal level (Tegeder et al., 2004; Tang et al., 2007; Quintero et al., 2011). Specifically, it has been reported that NO metabolites were represented as markers of postsynaptic NMDA receptor activation (Quintero et al., 2011). Thus, we speculated that NO-mediated SIH in our studies might respond via the activation of NMDA glutamate receptor, which induced the production and release of NO and in turn modulated presynaptic neurotransmitter release. However, this intriguing issue is required to be answered in the following studies.

Nitric oxide, a soluble gas, as a retrograde messenger modulating the release of various neurotransmitters, is associated with nociception in the peripheral and central nervous systems. NO via the production of constitutive NOS and iNOS could be observed in nervous tissues (Chu et al., 2005; Tang et al., 2007). Previous research suggested that BH4, a cofactor for NOS, could regulate the expression of NOS to produce NO (Tegeder et al., 2006). Remarkably, we showed that SIH induced up-regulation of iNOS, instead of nNOS and eNOS, in SIH mice. DAHP attenuated the pain threshold and down-regulated the expression of iNOS mRNA and protein. Besides, intrathecal treatment of 1400 W (a specific iNOS inhibitor) and 7-NI (a specific nNOS inhibitor) significantly attenuated the hyperalgesia evoked by exposure of RS (Tang et al., 2007), while only a slight decrease in NO metabolites’ accumulation is observed in 7-NI-treated SIH mice. These findings corroborate previous reports, indicating that nNOS might compensate for the function of iNOS in SIH (Tao et al., 2003). Nevertheless, our study did not directly clarify the possible role of eNOS in SIH due to the absence of available highly selective inhibitors of eNOS. Thus, we speculated that a substantial amount of NO via iNOS mainly modulated the SIH in chronic RS. These results are consistent with some previous literature (Olivenza et al., 2000; LaBuda et al., 2006; Tang et al., 2007), but contradictory to other reports (Kishimoto et al., 1996; Costa et al., 2005; Tang et al., 2007). The discrepancy might be due to the duration and intensity of stress models. It has been highlighted that iNOS is specifically co-localized with glia, macrophages, and neutrophils in various preclinical models after the stimuli of cytokines, microbial products, or lipopolysaccharide (Moncada et al., 1991; Gross and Wolin, 1995). Under chronic RS conditions, astrocytes in the superficial layer of the spinal dorsal horn become reactive, thus altering morphology and increasing the expression of GFAP, which is widely used as a marker of astrocytes. Moreover, we reported that iNOS was co-expressed with GFAP in the spinal cord instead of Iba1 and NeuN. To the best of our knowledge, there is no study reporting the cell type expressing iNOS in spinal. We speculated that activated astrocytes release a variety of pro-inflammatory cytokines (e.g., tumor necrosis factor-α and interleukin-1β) and neurotransmitters (e.g., NO) (Frank et al., 2007), which, therefore, induces neuronal sensitization, and future detailed study on the role of iNOS induced by BH4 in chronic RS model is required. In summary, the iNOS–NO cascade system partly mediated the SIH at the spinal level. Nevertheless, the underlying mechanisms of iNOS activation responsible for the spinal cord’s neurodegenerative changes are needed to clarify in further studies.

Finally, our study indicates the methylation locations of GCH1 in preclinical studies and, thus, may propose a more effective pain therapeutic approach and potential targets for analgesic drugs. Preclinical studies also show that BH4 inhibition with GCH1 by DAHP might occur through competition for the substrate GTP-produced antinociception effects in rats exposed to repeated RS, and BH4 enhances nociceptive responses partly mediated by prevention of excess NO production, suggesting that BH4 has a crucial role in the SIH. Further investigation is essential to understand the precise mechanisms of the BH4 pathway in SIH conditions, as it is reported that chronic RS induces hyperalgesia in male rats instead of female rats (Gamaro et al., 1998). The intriguing issue is required to be answered in further studies. Based on the current findings, our studies may contribute to a better understanding of chronic pain and may provide a more theoretical basis for the therapeutic drug approach in chronic pain.

## Materials and Methods

### Animal Grouping and Treatment

Pathogen-free, male Sprague–Dawley (SD) rats, weighing 190–220 g, and supplied by the Experimental Animal Center of Hubei Province, Tongji Medical College, Huazhong University of Science and Technology (HUST), were housed under a standard temperature (22 ± 2°C) room with standard rodent chow and water available *ad libitum*. All experiments were performed under a protocol approved by the Animal Care and Use Committee of HUST and were conducted following the National Institutes of Health Guide and Ethical Issue of the International Association for the Study of Pain. The animals were subjected to a 12-h light/dark cycle (lights on at 07:00 AM and off at 07:00 PM) maintained under constant conditions for 7 days before the experiment.

The animals were randomly assigned to nine groups: (1) control group (*n* = 12) with no intervention; (2) RS group (*n* = 12), in which 6 h (9:00 AM to 03:00 PM) of RS was conducted; (3) vehicle/RS group (*n* = 8), in which 20 μl of normal saline (NS) was delivered via an intrathecal catheter 15 min before RS; (4) BH4/RS group (*n* = 10), in which 10 μl (1 μg/μl) of BH4 was given and 10 μl of NS was used for flushing; (5) DAHP/RS group (*n* = 8), in which 10 μl (6 mg/kg) of DAHP was injected with 10 μl of NS flushing the PE-10 tube; (6) L-NAME/RS group (*n* = 9), in which 10 μl (30 μg/μl) of L-NAME was injected with 10 μl of NS; (7) L-NAME/BH4/RS group (*n* = 10), in which 10 μl (30 μg/μl) of L-NAME was injected 30 min before BH4 (1 μg/μl, 10 μl) was injected; (8) 1400 W/RS group (*n* = 10), in which 10 μl (1 μg/μl) of specific inhibitor of iNOS, 1400 W, was injected into the subarachnoid space with 10 μl of NS flushing the tube; and (9) 7-NI/RS group (*n* = 10), in which 10 μl (40 μg/μl) of specific inhibitor of nNOS, 7-NI, was injected into the subarachnoid space.

### Drugs

Tetrahydrobiopterin, 7-NI, 1400W, L-NAME, DAHP, and dimethyl sulfoxide (DMSO) used in the research were purchased from Sigma–Aldrich Co., United States. BH4 and L-NAME were dissolved in NS and administrated by intrathecal injection. 7-NI, 1400 W, and DAHP were first dissolved in DMSO and then diluted with NS to the desired concentration. All drugs were prepared immediately before administration and given in a volume of 10 μl with 10 μl of NS flushing the PE-10 tube. The dosage was based on Sung et al. (2004), Tegeder et al. (2006), and Makuch et al. (2013).

### Intrathecal Catheter Implantation

The method for intrathecal catheter implantation followed the steps in the previous laboratory reports (for details, see Supplementary Material) (Ke et al., 2013; Guan et al., 2015).

### Restraint Stress Model

**FIGURE 8:**
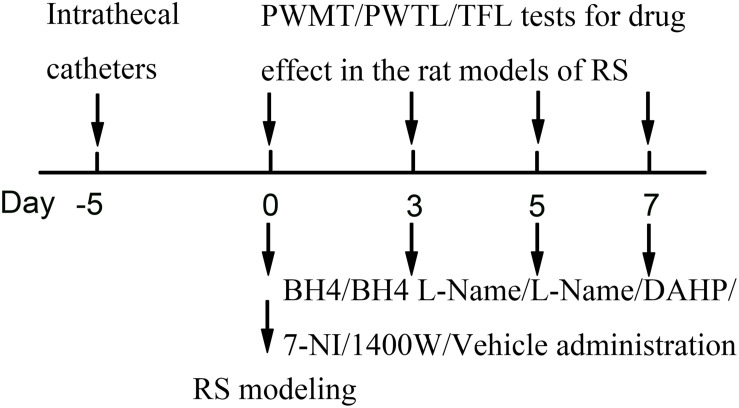
Experimental design. RS, restraint stress; PWMT, paw withdrawal mechanical threshold; PWTL, paw withdrawal thermal latency; TFL, tail-flick latency; BH4, tetrahydrobiopterin; L-NAME, *N*^ω^-nitro-L-arginine methyl ester; DAHP, 2,4-diamino-6-hydroxypyrimidine; 7-NI, 7-nitro indazole; 1400W, *N*-([3-(aminomethyl)phenyl]methyl)ethanimidamide.

Restraint stress was performed according to a previously described chronic RS model. The chronic RS was performed using a plastic tube (18 × 5 cm). Male SD rats (190–230 g) in the RS group were arranged on a plastic tube, leaving enough vents, with an iron clamp from outside penetration clamped tails, in order to adjust the position, avoiding visible physical damage for 6 h (from 09:00 AM to 03:00 PM) (Magarinos and McEwen, 1995). The non-RS animals were maintained in their home cage. Food and water were removed during the time that the RS rats were kept in the plastic tube. Drug administration was 15 min before the RS. Rats were repeatedly exposed to daily RS for seven consecutive days and put back to the home cage with standard rodent chow and water available *ad libitum* (Figure 8).

### Behavioral Assessments

Nociceptive tests such as the PWMT test, PWTL, and tail immersion test were performed as literature previously (for details, see Supplementary Material) (Taliyan and Sharma, 2012; Deciga-Campos et al., 2016).

### Western Blot Analysis

Western blot analysis was performed as reported previously (for details, see Supplementary Material) (Tegeder et al., 2006; Guan et al., 2015).

### Quantitative Real-Time Reverse Transcription–Polymerase Chain Reaction

The qRT-PCR analysis was performed as reported previously (for details, see Supplementary Material) (Chen et al., 2014).

### Immunofluorescence and Immunohistochemistry

Immunofluorescence and immunohistochemistry were performed as reported previously (for details, see Supplementary Material) (Guan et al., 2015).

### Nitric Oxide Production Assay

Immunofluorescence and immunohistochemistry were performed as reported previously (for details, see Supplementary Material) (Prast and Philippu, 2001).

### Measurement of the Tetrahydrobiopterin in the Spinal Cord and Plasma

High-performance liquid chromatography analysis was performed as reported previously (for details, see Supplementary Material) (Fukushima and Nixon, 1980; Matei et al., 2006; Fekkes and Voskuilen-Kooijman, 2007).

### GTP Cyclohydrolase 1 Methylation

GCH methylation analysis was measured as reported previously (for details, see Supplementary Material) (Li et al., 2012).

### Statistical Analysis

All statistical analyses were conducted using SPSS version 19.0 software (Chicago, IL, United States) and presented as the mean ± standard error of the mean (SEM). Data from the western blot, RT-PCR assays, etc., were analyzed using Student’s *t*-test to compare two groups or one-way analyses of variance (ANOVAs) for multiple comparisons followed by Bonferroni *post hoc* test or Dunnett’s T3 tests, if necessary. Data from the nociceptive behavior tests were evaluated using two-way ANOVA with repeated measures, followed by Bonferroni *post hoc* test or Dunnett’s T3 test, if necessary. *P* < 0.05 was considered statistically significant.

## Data Availability Statement

The original contributions presented in the study are included in the article/Supplementary Material. Further inquiries can be directed to the corresponding author/s.

## Ethics Statement

The animal study was reviewed and approved by the Animal Care and Use Committee of HUST.

## Author Contributions

YH and HX designed the study and wrote the protocol. BJ, BZ, BX, PL, LA, NY, and YZ performed all the experiments. YH, BJ, and HX undertook the statistical analysis and wrote the first draft of the manuscript. All authors have approved the final manuscript.

## Conflict of Interest

The authors declare that the research was conducted in the absence of any commercial or financial relationships that could be construed as a potential conflict of interest.
