# Supplementary material for: Nitric Oxide in the Spinal Cord Is Involved in the Hyperalgesia Induced by Tetrahydrobiopterin in Chronic Restraint Stress Rats
Source: Front Neurosci. 2021 Mar 26;15:593654. doi: 10.3389/fnins.2021.593654 (PMC8044835; doi:10.3389/fnins.2021.593654)
Supplement: Supplementary file 1 [file Data_Sheet_1.PDF]

frontiers

Supplementary Material

Supplementary Data

Intrathecal catheter implantation

All surgical procedures were conducted under abdominal anesthesia with pentobarbital sodium (40 mg/kg, i.p.). Pathogen-free male adult SD rats were placed on the surgical stage and fixed limbs after the rat anaesthetized adequately. The methods for catheter preparation and insertion were according to the method of previous reports of our laboratory(Ke et al., 2013; Guan et al., 2015). A longitudinal median incision about 1.0 cm located in L4-5 was performed. In turn, open the skin, subcutaneous fascia, exposure of the muscle layer, in the process of the spine. Separate the spinous process of L4-5 on the side of the knife back muscle with blunt, expose white lamina, and then gently insert the polyethylene catheter (PE-10 tube, with an inner diameter of 0.3 mm and outer diameter 0.6 mm, PE-0503, Anilab Software & Instruments, Ningbo, China) with tweezers from the facet joint medial of L4-5. When you insert into about 0.5 cm, if you can see the tail vacillate to the left and right and clear cerebrospinal fluid outflow from the PE-10 tube, then fixed the catheter on the subcutaneous fascia and the other end of the catheter fixed through the subcutaneous tunnel. Proper location of the catheter might induce at a temporary motor block of both hind-limbs after injection of 2% lidocaine 10 μl, followed by NS. Only the rat showed no evidence of neurologic deficit or paralysis can be studied for the following analysis.

Behavioral Assessments

Here we use three different nociceptive tests to evaluate the pain threshold, mechanical paw withdrawal threshold test (PWMT), thermal paw withdrawal latency (PWTL) and tail immersion tests, respectively. All the rats were arranged to a new environment for 30 min to adapt the environment. The experimenters were blind to the groups and the tests were conducted on the day before RS, and on the 3rd, 5th, 7th day at 15: 00 PM when finishing the RS.

1) The PWMT

Rats were placed on a metal mesh floor and covered with a transparent plastic dome 30 min prior to the study. They rapidly rest in the new environment after an initial period of exploration. Nine filaments (1-, 1.4-, 2-, 4-, 6-, 8-, 10-, 15-, and 26- g bending force, Stoelting, Wood Dale, IL, USA) were applied in the study, in ascending order of force for up to 3-4 s per filament. A positive response was the rats appearing the actions of licking or flinching the claws. When rats appeared positive responses, we may applied a lower force filament until no responses. Each monofilament was applied five times with a 15 s interval and the sensitivity of the mechanical stimuli to the filament bending force was recorded, in which at least 60% of the application induced responses were recorded.

2)The PWTL

The PWTL was assessed by using a thermal planter analgesia instrument (Ugo Basile 37370, Italy). In our laboratory, we change the intensity of the infrared heat (I.R.) to 90 and cut time to 20 s to

avoid causing obvious tissue damage. In the experiments, we posited the corner of the infrared source beneath the mid-plantar surface of the hind paw. We recorded PWTL when the rats licking the paws and the machine stopped recording the time and at least repeated 3 times with an interval of about 30 s between stimuli. The PWTL was expressed as a percentage of the maximum possible effect (%MPE).

$\%\mathrm{MPE} = (\mathrm{Post} - \mathrm{treatment\ latency} - \mathrm{Pre} - \mathrm{treatment\ latency}) \times 100 \div (\mathrm{Cut\ off\ time} - \mathrm{Pre} - \mathrm{treatment\ latency})$ (Taliyan and Sharma, 2012)

3)Tail flick latency(TFL)

A water-bath was utilized to assess the TFL. Once complete the period of RS, a 3 cm length of the distal tail was immersed into a heated water bath (50 ± 0.5 °C). In our study of the measurement, we utilized 49 °C for the better temperature, which may not causing tissue damage and the rat could recover quickly. A positive response was the rat violently jerking the tail out of the water and we recorded the time as the threshold. If there was no response, we sat up the cut time of 12 s to avoid obvious tissue damage(Deciga-Campos et al., 2016). Reaction latency (s) was used as a parameter reflecting the intensity of the pain experienced. Tail-flick results were expressed as % Analgesia.

$\%\mathrm{Analgesia} = (\mathrm{Post} - \mathrm{treatment\ latency} - \mathrm{Pre} - \mathrm{treatment\ latency}) \times 100 \div (\mathrm{Cut\ off\ time} - \mathrm{Pre} - \mathrm{treatment\ latency})$ (Taliyan and Sharma, 2012)

Western blot analysis

Western blot were analysis as previous reported(Tegeder et al., 2006; Guan et al., 2015). On the 7th day, four RS rats, four control rats, four BH4 / RS rats and four DAHP / RS rats were deeply anesthetized with pentobarbital sodium (60 mg/kg, i.p.). The tissues of the lumbar enlargement of the spinal cord at L2-5 were quickly moved and homogenized in RIPA lysis buffer (50 mmol/L Tris-HCl, pH 7.5, 0.5% DOC, 1% NP40, 0.1% SDS, 150 mmol/L NaCl, 1 mmol/L NaF, 1 mmol/L Na_3VO_4) supplemented with protease inhibitors (5 mM PMSF, 10 μg/mL leupeptin and 10 μg/mL aprotinin, all from Boster) on ice. The homogenates were shaked at 4 °C followed by centrifugation at 12,000 × g for 15 min. The concentration of the protein was quantitated by the BCA Protein Assay (Boster, AR0146, Wuhan, China). Samples were added the 5 × loading buffer (Boster, Wuhan, China) till the concentration was equal volume and quality. Then denature at 95 °C for 10 min, cool down to the RT and stored in -20 °C. Samples (50 μg of total protein per lane) were subjected to 10% SDS-polyacrylamide gel (PAGE) electrophoresis and transferred onto polyvinylidene fluoride (PVDF) membranes (IPVH00010, Millipore, Bellerica, MA, USA). After blocking in 5% BSA in Tween 20 / Tri-buffered saline (TBST, 0.1%) for 2 h at room temperature (RT, 23 ± 2 °C), the membranes were incubated overnight at 4 °C with primary antibodies against GCH1 (1:300, 25-0680, Abbiotec, San Diego, USA), nNOS (1:500, A1485, ABclonal, Wuhan, China), iNOS (1:1000, A0312, ABclonal, Wuhan, China), eNOS (1:500, A1548, ABclonal, Wuhan, China), β-actin (1:500, BM0626, Boster, Wuhan, China).The membranes were washed with TBST, then incubated with the second antibody conjugated with horseradish peroxidase-conjugated goat-anti-mouse (1:5000, BA1050, Boster, Wuhan, China) or goat-anti-rabbit secondary antibody (1:5000, BA1054, Boster, Wuhan, China) for 2 h at RT. A ECL Plus Kit (Beyotime, BeyoECL Star, poo18A, Wuhan, China) was used to exposed and a computerized image analysis system (BIO-RAD, ChemiDoc XRS+, CA, USA) to measure the signal.

Quantitative real-time reverse transcription-polymerase chain reaction (qRT-PCR)

The tissues isolated from the lumbar L2-5 were cut into pieces and placed in individual tubes on ice. For total RNA isolation, samples were thawed at RT and RNAiso Plus (1 ml / 50-100 mg sample, Takara, Japan) was added to the tubes to homogenize the samples. The operation was according to the manufacturer's protocol. The concentration of the total RNA was detected by a NanoDrop ND-1000 Spectrometer (NanoDrop Technologies). The primer of nNOS (RQP 051506), iNOS (RQP 048946), eNOS (RQP 050279), GCH1 (RQP 050796), GAPDH (RQP 049537) was synthesized by GeneCopoeia, America. 20 ng of cDNA from the same cDNA batch was added to Real-Time PCR to amplify all genes in triplicate in a total reaction volume of 20µl. A StepOne Plus™ Real-Time PCR System was used to conducted the reactions. The comparative threshold (Ct) method ($2^{-\Delta\Delta Ct}$) was used to calculate the relative gene expression(Chen et al., 2014).

Immunofluorescence and immunohistochemistry

Immunofluorescence and immunohistochemistry was performed as reported previously(Guan et al., 2015).On the7th day after the process of RS, three control rats and three RS rats were anesthetized with an overdose of pentobarbital sodium (60 mg/kg, i.p.) and perfused with 200 ml ice cold saline followed by 500 ml cold 4% paraformaldehyde (PFA, 0.1 M phosphate buffer, pH 7.4). After perfusion, the enlargement of the spinal cord (L3-5) were collected and postfixed in the 4% PFA in PBS for 24 h at 4 °C, and were subsequently dehydrated in 30 % sucrose solution for 48 h. Spinal cord transverse sections (20-µm-thick) were cut on a cryostat (CM1900, Leica, Wiesbaden, Germany). After washing in PBS for three cycles of 5 min each, transverse sections were penetrated with 0.3 % TritonX-100 for 15 min at RT to increase membrane permeability and blocked with 5 % bovine serum albumin for 1 h at RT, followed by incubation with rabbit anti-iNOS antibody (1:100, GB11119, rabbit anti-iNOS, Servicebio Technology CO., LTD, Wuhan, China) for 24 h at 4 °C. Then the sections were washed with PBS for 3 times of 5 min each and incubated with Fluoresceinisothiocyanate (FITC)- conjuguated donkey anti-rabbit secondary antibody (1:200, A24421-1, Abbkine, California, USA) for 2 h. To further identify the cell type expressing iNOS in chronic RS rats, primary antibody for this protein was co-cultured with primary antibody against mouse anti-glial fibrillary acidic protein (anti-GFAP, astrocyte biomarker, 1:400, MAB360, Merck Millipore, Darmstadt, Germany), or goat anti-Iba1(microglia marker, 1:300, ab5076, Abcam, Cambridge, UK) or mouse anti-NeuN (neuronal biomarker, 1:200, MAB377, Merck Millipore, Darmstadt, Germany). Second antibodies used were Cy3 donkey-anti-goat Ig (1:300, AS036, ABclonal, Wuhan, China), IFkine red AffiniPure donkey-anti-mouse Ig (1:300, A24411-1, Abbkine, California, USA). After being washed in PBS for 3 times, sections were mounted with Fluoromount-G solustion (0100-01, SouthernBiotech, Birmingham, AL, USA). Images were captured using a laser scanning confocal microscope (FV1000, Olympus, Tokyo, Japan).

Nitric oxide production assay

Due to a very low half-life (few seconds) and high reactivity, it is very difficult to directly quantify NO. But the content of the stable metabolites (nitrite and nitrate) of Nitric oxide is regarded as an index of NO formation, so in our study, we utilized the Nitrate reductase method to quantify NO. In vivo, NO was active and metabolize into nitrite ($NO2_-$) and nitrate (NO3-). The NO levels can accurately represented by the concentration of nitrite and nitrate in vivo. A NO assay kit (A012, Nanjing Jiancheng Bioengineering Institute) was used to measure NO concentration. The L2-5 lumber tissues in -80 °C were grinded into tissue homogenate supernatant and conducted according

to the assay kit. The absorbance of samples was determined at the light wavelength of 550 nm, diameter of 0.5 cm. The NO content (μmol/gprot) was calculated according to the formula, where the standard concentration is 20 μmmol/L(Prast and Philippu, 2001).

$$\mathrm{NO} = \frac{(\text{the sample value} - \text{the blank value})}{(\text{the standard value} - \text{the blank value})} \times \frac{\text{the standard concentration}}{\text{the sample protein concentration}}$$

Measurement of the BH4 in spinal cord and plasma

Homogenized spinal cords of rats, the plasma(Voskuilen-Kooijman, 2007) from cardiac blood sampling were obtained on the 7th day(Wang et al., 2016), BH4 and total biopterin concentrations were measured using a high performance liquid chromatography (HPLC) system and fluorescence detector as Matei *et al.* mentioned**(**Matei et al., 2006). Total biopterin represented the sum of BH4, BH2, biopterin BH4 and BH2 were oxidized to biopterin in acid conditions, whereas under iodine conditions, BH2 was still oxidized to biopterin, but BH4 to pterin. Thus, the difference in biopterin obtained from acid and iodine oxidation can used to calculated the BH4 level**(**Fukushima and Nixon, 1980).

GCH1 Methylation

The GCH1 gene sequence (5000 bp upstream of location of TSS (G) to 1000bp downstream of the last exon) was download from the NCBI and predicted the distribution of CpG islands using the CpG island searcher online software. We used the sequenom® EpiDesigner software to design the primer sequence and programs 21# and 22# (**Table 1**) were the recommended scheme. The genomic DNA was isolated using the TIANamp Genomic DNA Kit according to the manufacturer's specifications. Quantitative methylation analysis of the CpG islands were performed using the Sequenon MassArray platform (CapitalBio, Beijing, China) as previously described(Li et al., 2012). The location described in our study were using the Gene version number RGSC5.0/rn5.
